# Decreased endothelin receptor B expression in large primary uveal melanomas is associated with early clinical metastasis and short survival

**DOI:** 10.1038/sj.bjc.6600620

**Published:** 2002-11-12

**Authors:** S L Smith, B E Damato, A G M Scholes, J Nunn, J K Field, J Heighway

**Affiliations:** Gene Function Group, Roy Castle International Centre for Lung Cancer Research, 200 London Road, Liverpool L3 9TA, UK; Ocular Oncology Service, St Paul's Eye Unit, Royal Liverpool University Hospital, Liverpool L7 8XP, UK; Unit of Ophthalmology, Department of Medicine, University of Liverpool, Liverpool L69 3BX, UK; Molecular Genetics and Oncology Group, Clinical Dental Sciences, University of Liverpool L69 3BX, UK

**Keywords:** uveal melanoma, endothelin receptor B, comparative multiplex RT–PCR, metastasis

## Abstract

The most devastating aspect of cancer is the metastasis of tumour cells to organs distant from the original tumour site. The major problem facing oncologists treating uveal melanoma, the most common cancer of the eye, is metastatic disease. To lower mortality, it is necessary to increase our understanding of the molecular genetic alterations involved in this process. Using suppression subtractive hybridisation, we have analysed differential gene expression between four primary tumours from patients who have developed clinical metastasis and four primary tumours from patients with no evidence of metastasis to date. We have identified endothelin receptor type B as differentially expressed between these tumours and confirmed this observation using comparative multiplex RT–PCR. In a further 33 tumours, reduced endothelin receptor type B expression correlated with death from metastatic disease. Reduced expression also correlated with other known prognostic indicators, including the presence of epithelioid cells, chromosome 3 allelic imbalance and chromosome 8q allelic imbalance. Endothelin receptor type B expression was also reduced in four out of four primary small cell lung carcinomas compared to normal bronchial epithelium. We also show that the observed down-regulation of endothelin receptor type B in uveal melanoma was not due to gene deletion. Our findings suggest a role for endothelin receptor type B in the metastasis of uveal melanoma and, potentially, in the metastasis of other neural crest tumours.

*British Journal of Cancer* (2002) **87**, 1308–1313. doi:10.1038/sj.bjc.6600620 www.bjcancer.com

© 2002 Cancer Research UK

## 

Uveal melanoma (UM) is the most common primary intra-ocular malignancy in adults, affecting approximately six people per million of the population per year. Approximately 50% of all patients with uveal melanoma die of metastatic disease (Damato *et al*, 1996), despite successful treatment of the primary tumour. The main clinical features associated with poor survival are largest basal tumour diameter (LBTD), ciliary body involvement and extra-scleral extension. Tumours showing rapid regression following radiotherapy are also significantly more likely to metastasise (Glynn *et al*, 1989). Histologically, the most important factors associated with metastatic disease are the presence of epithelioid cells and microvascular patterns, such as closed loops (Folberg *et al*, 1993; 2001; Mäkitie *et al*, 1999).

Uveal melanomas tend to develop characteristic somatic mutations, particularly a loss of one copy of chromosome 3 (i.e. monosomy 3) and gains in chromosome 8q copy number (Prescher *et al*, 1996; Sisley *et al*, 1997; White *et al*, 1998; Scholes *et al*, 2001). These aberrations show a positive correlation with a poor prognosis for survival. Monosomy 3 shows a highly significant correlation with metastasis (Prescher *et al*, 1996; Sisley *et al*, 1997; White *et al*, 1998; Scholes *et al*, 2001). Prescher *et al* (1996) reported metastases from 17 out of 30 (57%) tumours with monosomy 3 compared to none out of 24 (0%) patients without monosomy 3, after a median follow-up of 3.4 years. The significant correlation of changes to chromosome 3 with metastasis suggests that genes on this chromosome may be involved in the metastasis of uveal melanoma. However, there have been few molecular genetic studies of uveal melanoma and there is very little information about the involvement of specific genes, on chromosome 3 or other chromosomes, in the progression to metastasis.

To identify genes involved in uveal melanoma metastasis we have performed suppression subtractive hybridisation (SSH). Using this technique, we have compared gene expression patterns between primary uveal melanomas with changes to chromosome 3, from patients with clinically evident metastasis, and melanomas without alterations to chromosome 3, from patients that have shown no evidence of metastatic disease (and whose tumours have favourable clinical and histological features). We describe here the identification of endothelin receptor type B (EDNRB) as a gene that is differentially expressed between primary uveal melanomas of different metastatic status and demonstrate the differential expression of EDNRB in a wider uveal melanoma series. We also show that EDNRB expression correlates with known predictive factors for survival and with the incidence of metastatic disease. Finally, we demonstrate that the down-regulation of EDNRB is not due to homozygous gene deletion.

## MATERIALS AND METHODS

### Patients and samples

Tumour and blood samples were obtained for experimental purposes. Informed consent was obtained from all patients, and the study approved by the Liverpool Research Ethics Committee. A sample of fresh tumour tissue, approximately 2–3 mm in diameter, was snap-frozen in liquid nitrogen. The remaining tumour was formalin fixed, paraffin-embedded and processed for routine pathological studies. Histological and clinical data were recorded in a computerised patient database. Allelic imbalance data was obtained by PCR-based microsatellite analysis using 10 microsatellite markers on the p and q arms of chromosome 3 (Nunn *et al*, manuscript in preparation). Allelic imbalance was recorded for informative markers if the intensity of a tumour allele was reduced by at least 30% relative to normal DNA. RNA samples from small cell lung carcinoma (SCLC) patients were generated as part of a wider, ethically approved, concurrent gene expression study being undertaken at the Roy Castle Centre.

Uveal melanomas were obtained from 41 patients, after primary treatment by enucleation (32 patients) or trans-scleral local resection (nine patients). The patients comprised 27 males and 14 females with a mean age of 64.1 years (range 29–85). The largest basal tumour diameter, measured by ultrasonography, averaged 16 mm (range 9.7–20.7) and the thickness averaged 9.2 mm (range 4–15). The ciliary body was involved in 23 eyes. Histological examination showed epithelioid cells in 28 tumours and closed loops in 27 tumours. The follow-up ranged to 4.28 years (mean 1.29, median 0.87). Metastatic disease occurred in 12 patients. Sixteen patients died. Twelve of these died of metastatic disease, with the other four deaths attributed to bronchial carcinoma, heart failure, brain haemorrhage, and myocardial infarction. Allelic imbalance at ten loci on chromosome 3 (indicating probable monosomy 3) was seen in 22 tumours and 21 tumours showed allelic imbalance at position 8q24.1.

### RNA and DNA extraction

Total RNA was extracted, from 20×20 μm sections of the frozen tumour samples, using TRIzol reagent (Invitrogen, Paisley, UK), following the manufacturer's protocol. RNA was run on formaldehyde agarose gels and on an Agilent 2100 Bioanalyser RNA chip (Agilent Technologies, Stockport, UK), to confirm its integrity. DNA was extracted from blood and 20×20 μm frozen tumour sections using standard procedures (Liloglou *et al*, 1997).

### Suppression subtractive hybridisation (SSH)

Double stranded cDNA was prepared from 2 μg of total RNA using the SMART™ PCR cDNA synthesis kit (Clontech, Cowley, Oxford, UK), following the manufacturer's protocol. SSH was performed using the PCR-Select™ cDNA subtraction kit (Clontech), again, following the manufacturer's recommended protocol. The resulting subtracted libraries were cloned into pCR®-Blunt II-TOPO® TA cloning vector (Invitrogen) and transformed into One Shot® TOP10 competent *E. coli* cells. Colonies from the subtracted libraries were randomly picked and grown in 96-well liquid cultures. Bacterial plasmids were isolated using the Qiagen Plasmid kit (Qiagen, Crawley, Sussex, UK), sequenced with T7 reverse primer, using the ABI PRISM® BigDye® Terminator Cycle Sequencing Ready Reaction Kit (Perkin Elmer, Warrington, UK), and run on an ABI 377 sequencer (Perkin Elmer).

### Comparative, multiplex RT–PCR

Expression of the endothelin receptor type B gene was investigated by comparative multiplex RT–PCR (cmRT–PCR). Reverse transcription was performed with a Reverse Transcription system kit (Promega, Southampton, UK), from 1 μg of total RNA, in a 20 μl synthesis. PCR reactions containing 1 μl of cDNA, four primers (0.5 μg each of the control and test gene primer pairs), 1×Taq reaction buffer (Roche, Lewes, East Sussex, UK), 200 μM dNTPs (Roche) and 1 unit of Taq polymerase (Roche), in a total of 50 μl, were assembled and cycled 30 times in a PE Applied Biosystems GeneAmp PCR System 9700 machine at 58°C for 1 min, 74°C for 1 min and 94°C for 1 min, with an initial denaturation of 2 min at 94°C and a final cycle of 58°C for 1 min, 74°C for 3 min. Reaction products were visualised on 2.5% agarose gels run in TAE, and analysed quantitatively by Agilent 2100 Bioanalysis using a DNA 500 test kit (Agilent Technologies), as directed. Control primers to HPRT (hypoxanthine-guanine phosphoribosyl transferase, HPRTF 5′-ACA CTT CGT GGG GTC CTT TTC A-3′ and HPRTR 5′-GCT GAC CTG CTG GAT TAC ATC A-3′) were used in uveal melanoma samples, and to hypothetical protein KIAA0228 (Accession number: D86981, HEF 5′-GAA CTG TGT GCA CTC CTA TTT G-3′ and HER 5′-CCG TGC CAA ATA CAC TGC ATG T-3′) which is levelly expressed in lung carcinomas, compared to matched normal lung tissue, in SCLCs. Primers to EDNRB were EDF 5′-GGA TGA AGC AAG CAG ATT CGC A-3′ and EDR 5′-TGC ATG CGA AAC GGT CCC AAT A-3′ and amplified a 383 bp product between exons 1 and 3. Primers were designed to be cDNA specific, and were confirmed as such by the absence of any specific amplification in a control genomic DNA reaction. Comparative multiplex RT–PCR involves the co-amplification, by PCR, of control and test sequences from cDNA, in the same reaction. Levels of a particular mRNA are compared between different samples, by relating expression levels of the test gene to those of a control gene, the expression of which is constant between samples.

### Comparative, multiplex PCR

Tumours were screened for amplification of the EDNRB gene by comparative, multiplex PCR (cmPCR). cmPCR reactions containing four primers (0.5 μg each of the control and test primer pairs), 1×Taq reaction buffer, 200 μM dNTPs (Roche) and 1 unit of Taq polymerase, in a total of 50 μl, were assembled and cycled 30 times in a PE Applied Biosystems GeneAmp PCR System 9700 machine at 58°C for 1 min, 74°C for 1 min and 94°C for 1 min, with an initial denaturation of 2 min at 94°C and a final cycle of 58°C for 1 min, 74°C for 3 min. Test primers to the EDNRB gene were EDF-G 5′-CTA CAC AGC ACT TCA GCT ACT G-3′ and EDR-G 5′-AAT GCA GCC ACA ACT CCA TGA G-3′. Control primers were to the β-globin gene (GLOF 5′-GCA GCT GAG TAG CAA GGA TG-3′ and GLOR 5′-ATC TGC ACA CTT GAG GGC ATG-3′. Products were visualised on 2.5% agarose gels.

### Statistical analyses

Spearman's correlation coefficient was used to assess the relationship between EDNRB expression and LBTD, both of which were continuous, non-parametric variables. The Mann–Whitney test was used to examine how EDNRB expression was associated with ciliary body involvement, epithelioid cells, closed loops, allelic imbalance on chromosome 3 and allelic imbalance on chromosome 8q. All *P* values were two-sided. A *P* value of ⩽0.05 was taken to indicate statistical significance.

Survival of patients according to the level of EDNRB expression was plotted according to the Kaplan–Meier method. Association between metastatic death and the level of EDNRB expression was assessed by Cox univariate analysis.

## RESULTS

### Identification of EDNRB as differentially expressed in metastatic uveal melanoma

In order to identify genes involved in the metastasis of uveal melanoma, we compared gene expression patterns between primary uveal melanomas from patients with and without clinically evident metastasis. Using SSH, cDNA pooled from four primary UM with monosomy 3, that had detectable metastases was compared with cDNA pooled from four UM without monosomy 3, and with no evidence of metastasis, within at least 3 years of diagnosis of the primary tumour. Subtractions were performed in both forward and reverse directions for the pooled sample pairs. That is, cDNA from each pool was used as tester and driver in two separate experiments, in order to identify cDNAs that were either over- or under-represented in the metastatic samples. Approximately 400 clones were obtained in each of the forward and reverse libraries. Of these, a total of 180 have been successfully sequenced, to date. Twenty of the sequenced clones (10 forward and 10 reverse) were prioritised for further investigation on the basis of several criteria, including a known role in metastasis, location to chromosomes 3 or 8 and the frequency of the sequence in the library. One of the prioritised clones identified in the reverse library (i.e. the library containing sequences under-represented in the metastasising tumours) matched part of the EDNRB gene as determined by a standard BLAST (Basic linear alignment search tool) search.

### EDNRB expression in uveal melanoma

**Figure 1:**
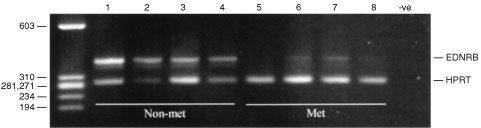
Comparative, multiplex RT–PCR amplification of EDNRB and the control gene HPRT in primary uveal melaonomas from patients with no evidence of metastasis (non-met) and patients with clinically evident metastasis (met).

**Figure 2:**
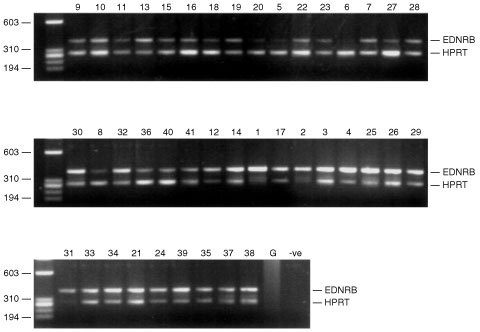
Comparative, multiplex RT–PCR of EDNRB and HPRT in uveal melanomas. G: genomic. –ve: Negative control. Numbers correspond to the tumour details in Table 1.

CmRT–PCR was used to verify the differential expression of EDNRB in the tumours used in SSH. Levels of EDNRB, relative to the control gene HPRT, were higher in the four primary tumours from patients with no evidence of metastasis compared to the four primary tumours from patients with clinically evident metastases (Figure 1

). No product was present in a negative control lane, nor in a genomic DNA control (see Figure 2

), indicating that the primers are cDNA specific. Agilent Bioanalysis of the cmRT–PCR results allowed quantification of the results (data not shown). The relative peak heights of each product were used to calculate the percentage expression of EDNRB relative to HPRT (see Table 1

**Table 1:**
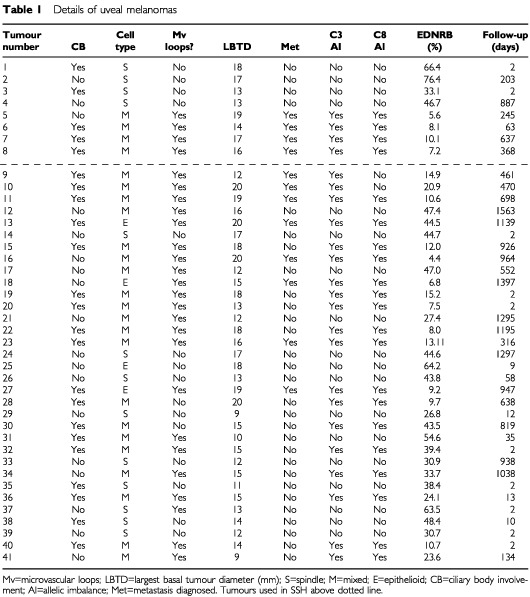
Details of uveal melanomas

). The level of EDNRB in the four tumours that had metastasised was between 4- and 50-fold less than the level of HPRT, whereas the level of EDNRB in the tumours that had no evidence of metastasis was approximately equal to the level of HPRT or greater (up to three-fold higher) than the level of HPRT.

### Association of EDNRB expression with other predictive factors for survival

To determine whether EDNRB expression could be correlated to metastatic disease and to other prognostic indicators, such as clinico-pathological variables and genomic alterations (specifically chromosome 3 loss), we analysed EDNRB expression in a further 33 uveal melanomas by cmRT–PCR (Figure 2). The relative percentages of EDNRB, compared to HPRT in each tumour, as determined by Agilent Bioanalysis are shown in Table 1, together with clinico-pathological data, genomic alterations and evidence of metastatic disease.

**Figure 3:**
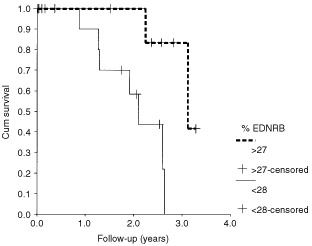
Relative percentage of EDNRB expression and survival.

The relative expression of EDNRB in the 33 tumours showed a non-normal distribution, with a median of 27.4% (range 4.4–76.4%). A relative percentage of EDNRB of less than 27.4% was defined as reduced expression. This cut-off point was selected *a priori* according to the median value. Reduced EDNRB was associated with allelic imbalance on chromosome 3 (*P*=<0.001), allelic imbalance on chromosome 8q (*P*=<0.001) and the presence of epithelioid cells (*P*=0.014). EDNRB expression did not correlate with LBTD (*P*=0.142), ciliary body involvement (*P*=0.07) or the presence of microvascular loops (*P*=0.07). Down-regulation of EDNRB was correlated with metastatic death (Figure 3

, Cox univariate analysis, *P*=0.05).

### EDNRB expression in SCLC

**Figure 4:**
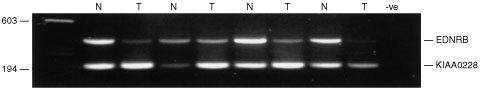
Comparative multiplex RT–PCR of EDNRB and the control gene KIAA0228 in SCLCs. N: cDNA from matched normal tissue. T: Tumour cDNA.

To determine whether EDNRB mRNA levels are reduced in SCLC we investigated expression of the gene by cmRT–PCR. Expression of EDNRB was analysed in cDNA derived from four primary SCLCs and in normal cDNA from the same patient. In all four cases the tumour cDNA showed reduced levels of EDNRB (relative to the control gene KIAA0228), when compared with cDNA from histologically normal lung tissue in the same patient (Figure 4

).

### Loss of EDNRB expression in uveal melanoma is not due to gene deletion

To investigate the underlying mechanism for reduced EDNRB expression in uveal melanoma, we examined whether the EDNRB gene on chromosome 13q22 was homozygously deleted. cmPCR was used to compare EDNRB copy number to that of the β-globin gene localised on chromosome 11p15. Reactions were balanced in normal DNA, from blood samples of patients with UM, to give approximately equal amounts of test and control products. The same ratio of control and test primers was then used in tumour samples. DNA from nine tumours was investigated. None of the nine tumours analysed (including four from patients with metastasis and five from patients with no evidence of metastasis) showed an imbalance between the test and control PCR products, compared to normal DNA from the same patient (data not shown).

## DISCUSSION

Previously, there have been very few investigations into the involvement of specific genes in the metastasis of uveal melanoma. Using suppression subtractive hybridisation, we have compared gene expression patterns between a pool of primary uveal melanomas from patients that have developed clinical metastases and a second pool of primary uveal melanomas from patients that have shown no evidence of metastasis. We have shown that endothelin receptor type B expression is lower in primary uveal melanomas of high metastatic genotype and phenotype than in tumours with no evidence of metastatic disease; an observation that has been validated in a wider series of tumours, using comparative multiplex RT–PCR.

In this study, reduced EDNRB expression correlated with monosomy 3 (as determined by allelic imbalance at multiple, chromosome spanning markers), changes to chromosome 8q (at position 24.1) and the presence of epithelioid cells. Reduced expression also correlated with metastasis-related death. The results of our analysis are significant because they show EDNRB down-regulation to be statistically related to known predictive factors and, indeed, to metastatic disease. EDNRB expression did not correlate with largest basal tumour diameter. This is likely due to the fact that there were few small tumours in our sample, most small tumours having been treated by phototherapy or radio therapy. Two patients developed metastatic disease despite having a normal EDNRB level. Mutations within the EDNRB gene, or within other genes in linked pathways, may explain this observation. Several patients with low EDNRB levels have not developed clinical metastases to date. Since uveal melanoma has a propensity for late clinical metastasis, sometimes up to 15 or 20 years after enucleation (Jensen, 1982), it is possible that these tumours have micrometastases even if no clinically evident metastases are apparent at the present time.

Metastasis describes the process by which cancerous cells escape the primary tumour, enter the bloodstream or lymphatics (in other cancers, but not in uveal melanoma) and form micrometastases at other sites in the body. These clonal lesions may subsequently grow into secondary tumours. Whether EDNRB down-regulation is associated with the initiation of metastasis (at the point where single cells escape the primary tumour), the survival of the detatched cells in the bloodstream or whether its down-regulation is associated with the establishment and growth of the secondary tumour remains to be determined. It is important to point out that due to the lack of small tumours in this study, our observations concerning EDNRB down-regulation and metastasis cannot be applied to small T1 and T2 uveal melanomas. Furthermore, we cannot determine at which stage EDNRB down-regulation occurs. Studies using smaller tumours, and uveal melanoma cell lines from tumours at different stages of progression, would go some way to determining at which stage down-regulation of EDNRB occurs.

It is also important to determine whether down-regulation of EDNRB occurs as a result of direct genetic damage (point mutations of control regions or the gene sequence itself, chromosomal translocations, gene deletions) or whether its expression is altered due to damage to other genes at higher points in control pathways. In a study by Aalto *et al* (2001), 50% of metastases from primary uveal melanomas were shown to exhibit losses in 13q, by comparative genomic hybridisation (CGH). We therefore investigated whether the observed reduced EDNRB levels were due to deletion of the EDNRB gene at 13q22. We did not detect any changes to the EDNRB gene copy number in any of nine tumours analysed. Whilst homozygous loss (HL) may be difficult to detect in primary tumour material, given that all the tumours used had tumour cell percentages in excess of 90%, if HL was present we would have expected the cmPCR to detect that loss. It would therefore appear that an alternative mechanism to gene deletion leads to the reduced expression of EDNRB. EDNRB is encoded by a gene that contains a 5′ CpG island encompassing the transcriptional regulatory region. This region has been reported to be methylated in 70% of prostate cancer samples (including primary tissue and cell lines) analysed by Nelson *et al* (1997) but unmethylated in normal tissues. CpG island methylation of the EDNRB gene promoter may therefore be responsible for the reduction in EDNRB levels in metastatic uveal melanoma.

Down-regulation of EDNRB occurs in other cancers. A comparison of EDNRB in four cutaneous melanoma cell lines originating from metastases, with three cell lines from primary tumours has shown the metastatic tumours to be characterised by low level expression of EDNRB (Kikuchi *et al*, 1996). Similarly, Grant *et al* (1997) have reported that human prostate cancer progression to metastases is accompanied by a reduction in EDNRB levels. In this study, we have also found reduced EDNRB levels in four out of four SCLCs, relative to matched normal lung control tissue. Similar to uveal melanoma, SCLC are neural crest derived tumours that exhibit changes to chromosome 3 (Sundaresan *et al*, 1992) and have a particularly aggressive clinical course, with frequent widespread metastases at diagnosis. However, not all studies have produced consistent results. For example, using immunohistochemistry and RT–PCR, Demunter *et al* (2001) reported increased EDNRB levels in metastatic cutaneous melanomas as compared with primary melanomas. The role of EDNRB in both uveal melanoma and other cancers, therefore, remains unclear.

In summary, we have shown that EDNRB shows reduced expression in large primary uveal melanomas of high metastatic genotype and phenotype, and in small cell lung cancer. The exact role of EDNRB in metastasis, the mechanism responsible for reduced expression and whether its down-regulation drives the metastatic process remain to be determined. EDNRB is a 7-span transmembrane G-protein coupled receptor, and since membrane-located receptors (mainly G-protein coupled receptors) constitute approximately 45% of all therapeutic drug targets (Drews, 2000), if a role in metastasis is proven, EDNRB, may be a good candidate for targeted therapy.
